# Deep ploughing in the summer fallow season and optimizing nitrogen rate can increase yield, water, and nitrogen efficiencies of rain-fed winter wheat in the Loess Plateau region of China

**DOI:** 10.7717/peerj.14153

**Published:** 2022-10-07

**Authors:** Rongrong Zhang, Peiru Wang, Wenxiang Wang, Aixia Ren, Hafeez Noor, Rong Zhong, Zhiqiang Gao, Min Sun

**Affiliations:** 1 Department of Agronomy, Shanxi Agriculture University, Taigu, Shanxi, China; 2 Collaborative Innovation Center for High-Quality and Efficient Production of Characteristic Crops on the Loess Plateau Jointly Built by Provinces and Ministries, Taigu, Shanxi, China; 3 Shanxi Agricultural University, State Key Laboratory of Sustainable Dryland Agriculture, Taiyuan, Shanxi, China

**Keywords:** Tillage methods, Soil water consumption, Plant nitrogen translocation, Grain yield, Water use efficiency, Nitrogen use efficiency

## Abstract

**Background:**

About 60% of the annual precipitation in the Loess Plateau occurs during the summer fallow season, and does not align with the wheat growing season. In addition, the nitrogen use efficiency is low in this area because nutrient availability is affected by drought. As a result, rainwater storage during the summer fallow season is very important to increasing nitrogen use efficiency, and to the stable production of dryland wheat in the Loess Plateau.

**Methods:**

A 3-year field experiment in the eastern part of the Loess Plateau was conducted with two tillage methods (no tillage (NT) and deep ploughing (DP)) and five N rates (0, 120, 150, 180, and 210 kg N ha^−1^) to study the effect of tillage on soil water utilization, plant nitrogen utilization, and wheat yield.

**Result:**

Compared to NT, DP showed a larger increase in soil water storage (SWS_f_) and precipitation storage efficiency (PSE_f_) during the two dry summer fallow seasons than in the normal summer fallow season. DP substantially increased the pre-anthesis soil water consumption (SWC_pre_) and N translocation. The average yield under DP was 12.46% and 14.92–18.29% higher than under NT in the normal and dry seasons, respectively. A 1 mm increase in SWC_pre_ could increase grain yield by 25.28 kg ha^−1^, water use efficiency (WUE) by 0.069 kg ha^−1^ mm^−1^, and nitrogen utilization efficiency (NU_t_E) by 0.029 kg kg^−1^. DP could reduce the N rate by 11.49–53.34% in the normal seasons and 40.97–65.07% in the dry seasons compared to the same highest point of yield, WUE, and NU_t_E under NT.

**Conclusion:**

Deep ploughing in the summer fallow season, paired with optimized N application, could help increase wheat yield and nitrogen efficiency in dryland.

## Introduction

Wheat accounts for 26% of total global agricultural production, making it one of the most important crops for meeting the daily needs of humans worldwide (FAO, 2020). In China, the wheat planting area accounts for approximately 22% of the total crop cultivating area, and the Loess Plateau is one of most important cereal-producing regions, with wheat production accounting for 44% of all cultivated land. Thus, maintaining stable winter wheat production is crucial on the Loess Plateau because it provides a primary staple food source for residents of the area. On the Loess Plateau, more than 60% of the precipitation occurs when the winter wheat fields are fallow (July to September), during which time evaporation rates are high because of high temperatures (Jin et al., 2007). Precipitation during the growing stage (October to June) is less than 30–40% of the annual precipitation and is not enough to be effectively used for the growth of winter wheat (Fang et al., 2010; Sun et al., 2011). This low abundance and uneven distribution of precipitation significantly impacts wheat production in the region. Stable and high yield of dryland wheat is closely related to soil water storage before sowing, and precipitation during the summer fallow season is conducive to the recovery of soil moisture (Unger, Payne & Peterson, 2006; Schillinger, Schofstoll & Alldredge, 2008; Xue et al., 2019).

Dry matter (DM) production and nitrogen (N) uptake are largely regulated by the available water in the soil (Plett et al., 2020). Tillage affects soil properties, which in turn affect crop root growth and grain yield (Chu et al., 2016; Xue et al., 2019). Previous studies have pointed out that areas without tillage suffer from significant soil compaction, leading to reduced water infiltration and limited root growth and crop yields (Peralta, Alvarez & Taboada, 2021). Tillage promotes microbial activity, which improves the availability of N in the soil (Soon et al., 2007; Ruisi et al., 2016). In a long-term study of the Loess Plateau, we showed that deep ploughing and subsoiling in the summer fallow season mitigates soil compaction by loosening shallow soil and breaking the plough pan, increasing soil water storage in the 0–300 cm layer by 8% (Sun et al., 2018; Xue et al., 2019). Higher soil water content can be obtained through deep tillage and subsoiling during different crop development stages (Shi et al., 2016). At different stages, the soil layer with the greatest water consumption gradually deepens, and the correlation between water consumption and yield after anthesis is higher at depths of 180–240 cm than in the shallower layers (Xue et al., 2019). Furthermore, when compared with the no tillage (NT), deep ploughing significantly increased water use efficiency, yield, and aboveground plant DM at maturity. Jia et al. (2004) reported that reduced tillage had no effect in the first 3 years but then decreased grain yield by 31.83% in subsequent years. Another study found that NT increased the soil temperature, soil moisture, and total crop yields, but reduced the soil respiration of wheat and maize under straw mulching (Li et al., 2022).

Tillage also significantly influences DM production and allocation of N to plant organs. As the most necessary mineral nutrient for plants, nitrogen plays an important role in increasing crop yield and the quality of agricultural products. A desire for increased crop yield is leading to a strong demand for N addition to fields worldwide (Khan et al., 2014; Schils et al., 2018). However, a certain amount of N is lost from fields in cropping systems, as a result, only around 47% of the applied N is efficiently used (Ladha et al., 2016; Swaney, Howarth & Hong, 2018). This lost N can leach as nitrate-N into groundwater, or release nitrous oxide into the air, both of which result in harmful impacts to the environment (Fowler et al., 2013). Thus, it is widely acknowledged that reducing nitrogen loss and improving nitrogen use efficiency are critical to crop production, environmental protection, and the development of sustainable agriculture (Raun & Johnson, 1999; Hirel et al., 2007). One study showed that 5 years after converting from tillage to NT, there were significant increases in N utilization efficiency and N remobilization under three N fertilization regimes (0, 161, 215 kg ha^−1^), but the grain yield and grain N content were similar under both CT and NT (Habbib et al., 2017). The one-time application of nitrogen fertilizer before sowing for dryland wheat is the main reason for the low nitrogen use efficiency, on the Loess Plateau, drought and water shortages also reduce the effectiveness of soil nutrients. If the nitrogen fertilizer input is too high, the vigorous growth of wheat before winter can aggravate the drought. If the nitrogen fertilizer input is too little, the wheat will not form strong seedlings before winter, resulting in insufficient nutrient supply in the later growth stages. Too much or too little nitrogen input will cause premature senescence in the later stage of growth and reduce yield and nitrogen use efficiency. Therefore, determining the appropriate amount of nitrogen fertilizer based on precipitation or soil water before sowing is important for improving both yield and water and nitrogen use efficiency. Proper tillage combined with an adequate N rate can decrease soil nitrogen loss and increase grain production while lowering N input (Zhou & Butterbach-Bahl, 2014). However, the effects of deep ploughing, compared with no tillage, on soil water consumption, plant growth, and nitrogen accumulation of winter wheat and the regulation of nitrogen inputs are largely unknown. In this study, we investigated the effects of tillage in the summer fallow season and nitrogen application rates on soil water consumption, dry matter and nitrogen translocation and total wheat yield and identified the best tillage methods and nitrogen rate during different precipitation seasons. Our objectives were: (1) to examine the effects of deep tillage on precipitation storage ability during the summer follow season as well as the soil water content at sowing and soil water consumption during plant growth in both dry and normal seasons; (2) to assess the effects of deep tillage and N rate on nitrogen balance of aboveground and soil, total grain yield, water use efficiency (WUE), and nitrogen utilization efficiency (NU_t_E) in both dry and normal seasons; and (3) to evaluate the contributions of the soil water storage amount and efficiency during the summer fallow season to soil water consumption, crop yield, and the optimization of the N rate on the basis of the yield, WUE, and NU_t_E in the dry and normal seasons.

## Materials and Methods

### Site description

Field experiments were carried out in a dryland at the Agriculture Research Station of Shanxi Agricultural University in Wenxi (35°20′N, 111°17′E), Shanxi Province, China, from July 2014 to June 2017. The experimental site is located in the northeast tableland of the Loess Plateau, where the dominant cropping system is winter wheat–summer fallow. In this region, over 60% of the precipitation falls in the fallow season (July to September). The experimental site is hill-dryland. From 1964–2014, the annual mean precipitation in the area was 529 mm and the annual mean temperature was 12.9 °C.

Weather data were collected and recorded by a weather station (AWS 800; Campbell Scientific, Inc., Logan, UT, USA) approximately 100 m from the experimental field. Precipitation type was defined as follows (Ren et al., 2019):

$\rm {P = (P_T-P_A)/P_A}$

$\rm Dry{:}\, P \leq -\!25{\%}$

$\rm Normal{:} -\!\!25{\%} \lt P \lt 25{\%}$

$\rm Wet{:}\, P \geq 25{\%}$

where P_T_ was the total precipitation of the summer fallow-winter wheat growth season *i.e*., July (previous year) to June (following year) and P_A_ was the thirty-season average precipitation (1988–2017: 486.8 mm).

**Figure 1:**
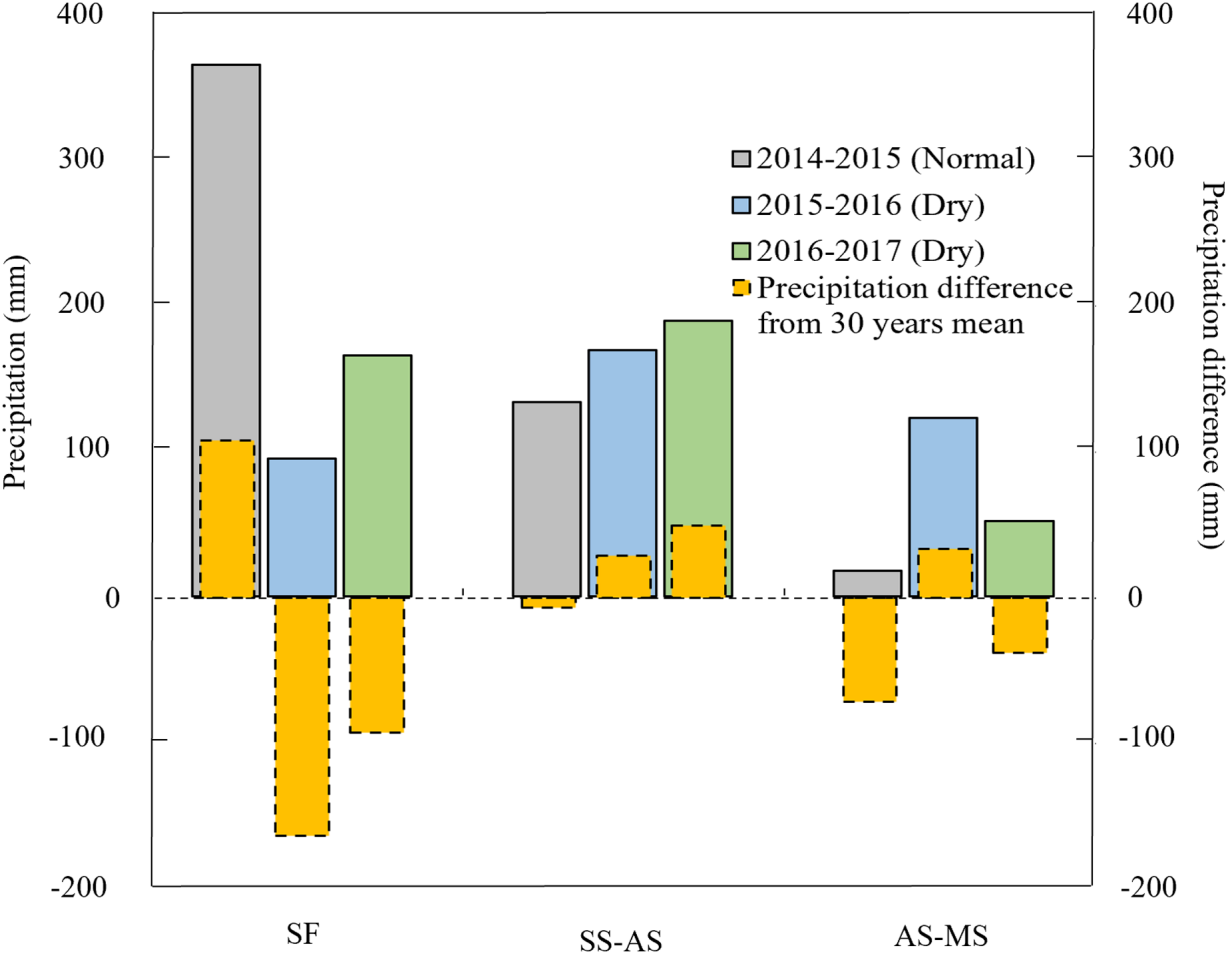
Precipitation during the summer fallow season and wheat growth period in 2014/2015, 2015/2016 and 2016/2017 and the difference from the 30-year precipitation average (from 1988 to 2017). Summer fallow (SF) is defined as June 21 to September 30, Sowing to anthesis (SS-AS) is October 1 to May 10 of the following year, and anthesis to maturity (AS-MS) is May 11 to June 20.

The annual precipitation in 2014/2015 was close to the thirty-season average, and therefore 2014/2015 is referred to as the “normal season” in this study (Fig. 1). The annual precipitation in 2015/2016 and 2016/2017 were 25.08% and 26.88% lower than the thirty-season average, so 2015/2016 and 2016/2017 are referred to as the “dry seasons” in this study (Fig. 1). Based on experiments in the Loess Plateau for eight consecutive seasons, Yu et al. (2021) classified the rainfall patterns in the summer fallow season and obtained similar results as ours in this study.

The soil at the experimental site was classified as calcareous cinnamon soil according to the classification defined by the International Soil Science Society (ISSS) (sand: 43.9%, silt: 32.0%, and clay: 10.2%). Before sowing in 2014–2015, basic soil properties were measured (Soil Survey Staff, 2010): the soil pH was 8.01, the total N was 0.53 g kg^−1^, the Olsen P was 12.56 mg kg^−1^, available K was 201.55 mg kg^−1^, and the soil bulk density was 1.29 g cm^−3^ (cutting-ring method, Xue et al., 2017).

### Experimental design and field management

An entire terraced field was divided into three parts. To avoid N and tillage effect buildup, a different field was used each year. The experiments used a split-plot design with three complete replications. Each 2.5 × 10 m plot (Fig. S1) used tillage methods in the summer fallow season and sub-plot treatments with five rates of N fertilizer at sowing (0, 120, 150, 180, and 210 kg N ha^−1^). A detailed description of the plot treatments is shown in Table 1.

**Table 1:** Description of tillage practice and nitrogen treatments.

Tillage methods	Practice during fallow period	Farming methods	Fertilizer before sowing	Cultivation methods
No tillage (NT)	No-tillage	In the end of August, rotary tillage and land leveling were conducted on 25 August 2014, 27 August 2015, and 28 August 2016 to remove weed and prepare the land of planting.	Nitrogen fertilizer (urea containing 46% N, 0, 120, 150, 180 and 210 kg N ha^−1^), P_2_O_5_ (150 kg ha^−1^), and K_2_O (90 kg ha^−1^) were applied once before sowing, and the detailed date same with the sowing date.	The winter wheat cultivar (Yunhan-20410) was sown on 25 September 2014, 23 September 2015 and 22 September 2016, and the seeding rate was 180 kg ha^−1^ by drilling method and row spacing was 20 cm.
Deep ploughing (DP)	DP was performed with furrow plough (TH-FZL-2, Tianhe machinery equipment factory, Jinan, China) at the depth of 30 cm on 12 July 2014, 10 July 2015, and 12 July 2016.			

To avoid yield losses, weeds were removed by hand, and pests and diseases were controlled during the main season using the conventional farming practices of the area. Plants were machine-harvested on 10 June 2015, 6 June 2016, and 8 June 2017.

### Plant sampling, measurement, and calculations

#### Soil moisture

Soil samples from a depth of 2 m were excavated using the cutting ring method at 20 cm depth intervals (Dama et al., 2005). Soil water storage (SWS, mm) was determined using the oven-drying method (Ma et al., 2015; Liu et al., 2016) on the 45th day after harvesting, at sowing, at anthesis, and at maturity. SWS was calculated as follows (Sun et al., 2018):

(1) $$\rm SWS = {BD/\rho w \times SWC \times H}$$

where SWS, BD, SWC, H, and ρw represent the soil water storage (mm), bulk density (g cm^−3^), water content (g water g^−1^ dry soil), depth (mm), and water density, respectively. BD and SWC were calculated according to the methods outlined by Sun et al. (2018).

Soil water consumption (SWC, mm) during the growing season was evaluated using the following formula:

(2) $$\rm SWC = R + \Delta SWS$$

where R (mm) is the total precipitation amount during the growing season and SWC_pre_, SWC_post_, and SWC_t_ (mm) are measurements of pre-anthesis, post-anthesis, and total crop soil water consumption.

Water storage efficiency during the summer fallow season (PSE_f_) was calculated as follows:

(3) $$\rm PSE_f = {SWS_f / P_f}$$

where SWS_f_ and P_f_ represent the soil water storage (0–200 cm) and precipitation during the summer fallow season, respectively.

#### Plant N accumulation and translocation

The N concentration of different organs and the total plant N accumulation were measured using the indophenol-blue colorimetric method (Meyer, 1983). N accumulation and translocation were calculated as followed (Moll, Kamprath & Jackson, 1982; Cox, Qualset & Rains, 1986; Spyridon, Sideris & Christos, 2012):

(4) $$\eqalign{&\rm Pre{-}anthesis\, N\, translocation\, (kg\, ha^{-1}) \cr&= \rm N\, at\, anthesis{-}N\, of\, vegetative\, parts\, at\, maturity } $$

(5) $$\eqalign{&\rm Contribution\, of\, pre{-}anthesis\, N\, translocation\, to\, grain\, ({\%}) \cr&= \rm (N\, translocation / grain\, N\, at\, maturity)\times 100 } $$

#### Soil and plant N balance and N uptake efficiency

At sowing and harvesting, a soil sample (approximately 100 g) was taken from each plot and then mixed and divided into two subsamples. The first sub-sample was analyzed for ${\rm NH}_4^ + {\rm \; }$ –N (Forster, 1995) and ${\rm NO}_3^ - {\rm \; }$ –N (Miranda, Espey & Wink, 2001). In the no-N (N0) plots, the apparent N mineralization rate (N_organic_) during the winter wheat growing season was calculated as follows (Cabrera & Kissel, 1988; Olfs et al., 2005):

(6) $$\rm apparent\, N\, mineralization\, rate\, (N_{organic},\, kg\, ha^{-1})= soil\, N_{0(start)}-soil\, N_{0(end)}$$

Apparent N losses (N_loss_, kg ha^−1^) were calculated in plots with N application using the following formula (Zhao et al., 2006):

(7) $$\rm N_{loss}=soil\, N_{(start)}+N_{fer} + N_{organic}- soil \,N_{(end)}-plant\, N$$

Soil N_(start)_ and _(end)_ represent the N content of the 0–100 cm soil profile at sowing and harvesting, respectively, and N_fer_ is the N rate.

(8) $$\rm N\, uptake\, efficiency\, (NU_{p}E, {\%}) = plant\, N\, accumulation/the\, N\, rate$$

#### Grain yield and water, nitrogen utilization

Plot grain yield was determined by harvesting all plants in a 20 m^2^ area from the center of each plot to eliminate marginal effect. The harvested grain was then shelled using an FM-600 machine (Qufu Fumin Machinery Manufacturing Co. LTD, Shandong, China) and then air-dried before measuring grain yield (grain moisture was adjusted to 12.5%).

WUE (kg ha^−1^ mm^−1^) was calculated as follows:

(9) $$\rm WUE=Y/ET$$

where Y is the grain yield (kg ha^−1^), and ET (mm) is the total evapotranspiration over the entire growing season using the following formula (Ren et al., 2019; Lin et al., 2019):

(10) $$\rm ET = P+\Delta SWS$$

where P and ΔSWS represent the total rainfall during the growing stage and the change in 0–200 cm soil water storage (planting to maturity), respectively.

(11) $$\rm N\, utilization\, efficiency\, (NU_{t}E,\, kg\, kg^{-1}) = grain\, yield /plant\, N\, accumulation \times 100{\%}$$

#### Data analysis

Statistical data analyses (analysis of variance) were performed in Statistix 8.0 (Analytical Software, Tallahassee, FL, USA). Differences of means of the treatments were compared based on the least significant difference (LSD) test with a probability of 0.05. Correlations between traits were obtained using Pearson’s correlation analysis in SPSS software v.26.0 (IBM Corp, Armonk, NY, USA).

## Results

### Effects of tillage on soil precipitation storage in summer fallow

In 2015/2016, 24.48% of the annual precipitation occurred during the summer fallow season, while in 2014/2015, 70.76% of the precipitation occurred during the summer fallow season. There was little difference in the precipitation amounts during the growing seasons of the 3 years, which ranged between 51.2 and 141 mm (Table 2). The normal season had the highest SWS_f_, and the PSE_f_ in the dry season was higher than in the normal season under DP, but lower under NT. Compared to NT, the SWS at sowing, SWS_f_, and PSE_f_ under DP were 7.65%, 38.87%, and 38.87% higher in the normal season, respectively, and 6.34–8.57%, 84.63–110.41%, and 84.48–110.83% higher in the two dry seasons, respectively.

**Table 2:** Effect of tillage on soil water storage and water storage efficiency during the summer fallow seasons in 2014/2015, 2015/2016 and 2016/2017.

Year	Tillage methods	P_f_ (mm)	Soil water storage (mm)			PSE_f_ (%)
			Pre-harvest	sowing	SWS_f_	
2014/2015	NT	365.6	391.3	499.6 b	108.3 b	29.62 c
(Normal)	DP			541.7 a	150.4 a	41.13 b
2015/2016	NT	94.7	363.2	385.3 e	22.1 e	23.31 d
(Dry)	DP			409.7 d	46.5 d	49.13 a
2016/2017	NT	165.4	375.1	417.4 d	42.3 d	25.63 cd
(Dry)	DP			453.2 c	78.1 c	47.28 a

**Note:**

No tillage (NT), and deep ploughing (DP). Precipitation during the fallow period (P_f_), soil water storage during summer fallow (SWS_f_), and precipitation storage efficiency during summer fallow (PSE_f_). Means within the same column with different lower letters were statistically significant at the critical probability levels of 0.05.

### Effects of tillage and N rate on the soil water consumption of winter wheat

**Figure 2:**
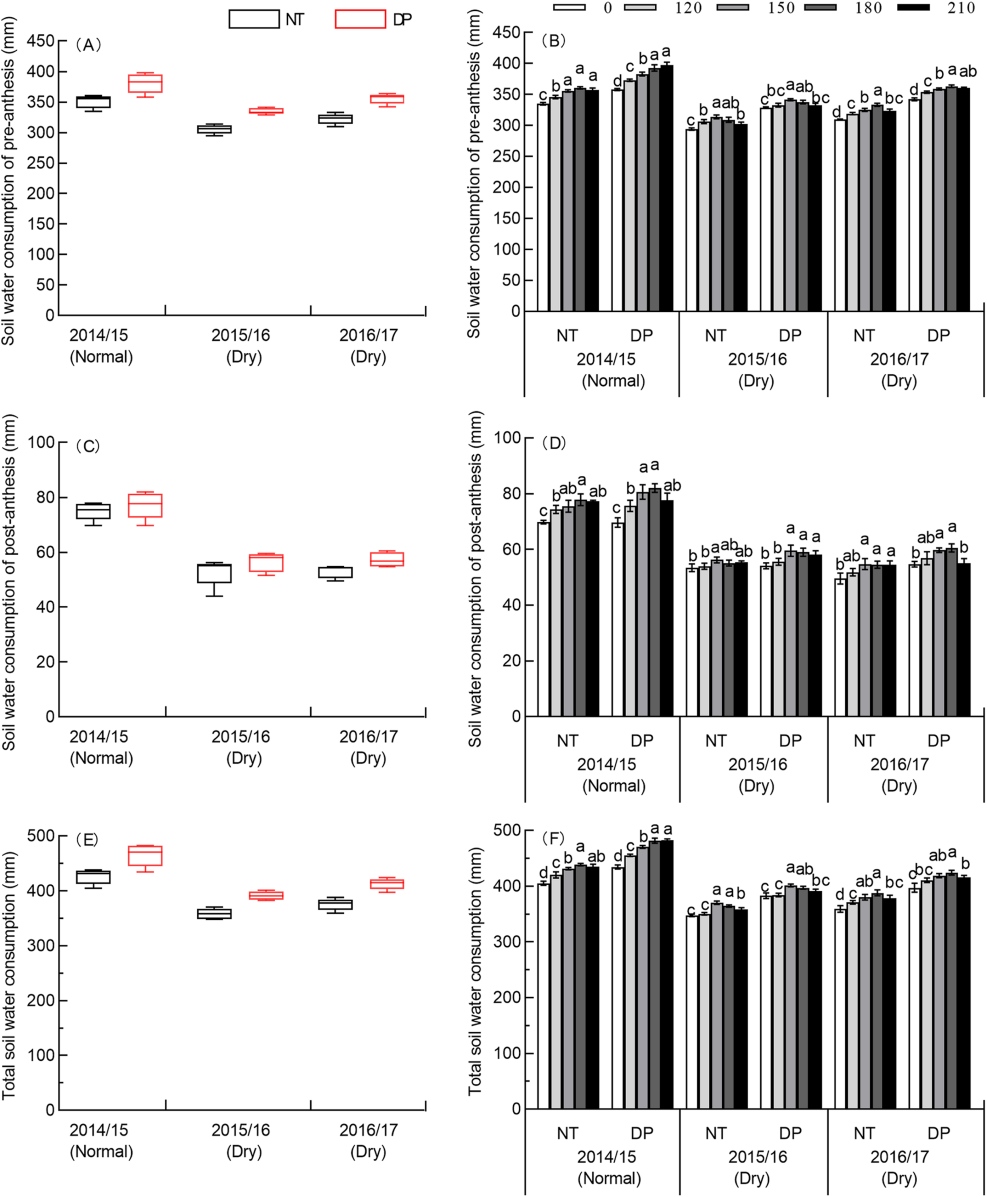
Effects of tillage and N rate on soil water consumption of 0–200 cm soil depth in three seasons. No tillage (NT), and deep ploughing (DP). Pre-anthesis soil water consumption (A), post-anthesis soil water consumption (C) and total soil water consumption (E) of two tillage in three seasons. Solid and red dashed lines represent median values, box boundaries represent the 25th and 75th percentiles, and bars and dots in or outside the boxes represent the 10th and 90th percentiles of all data. Pre-anthesis soil water consumption (B), post-anthesis soil water consumption (D) and total soil water consumption (F) of two tillage at five different N rates in three seasons. The means and SE of three replicates are presented. Different lowercase letters indicate significant differences between treatments at *P* < 0.05 by the LSD test.

Soil water consumption were affected by year, tillage, N rate, and the interactions of all these variables except the year × tillage and year × tillage × N rate on post-anthesis soil water consumption (Table S1). The average pre-anthesis soil water consumption in the normal season (2014/2015) was 12.11–12.99% higher than in the 2015/2016 dry season and 6.62–8.19% higher than in the 2016/2017 dry season (Table 2A). The average post-anthesis soil water consumption in the normal season was 26.82–29.51% higher than the 2015/2016 dry season and 25.65–29.28% higher than the 2016/2017 dry season (Fig. 2C). The average total soil water consumption in the normal season was 15.81–15.90% higher than the 2015/2016 dry season and 11.12–11.90% higher than the 2016/2017 dry season (Fig. 2E). There was a substantial increase in soil water consumption in the normal season due to the higher rainfall in the summer fallow season, and an increase of 286.06% over the 2015/2016 dry season and 121.04% over the 2016/2017 dry season.

Compared to NT, DP had a higher average soil water consumption, both pre-anthesis and post-anthesis. The average pre-anthesis soil water consumption for DP was 8.56% higher than NT in the normal season, and 9.67% and 10.42% higher than NT in the two dry seasons. The average post-anthesis soil water consumption for DP was 2.88% higher than NT in the normal season, and 6.81% and 8.16% higher than NT in the two dry seasons. The average total soil water consumption for DP was 9.13% higher than NT in the normal season and 9.25% and 10.10% higher than NT in the two dry seasons. Compared with NT, the increases in pre-anthesis soil water consumption under DP were higher than post-anthesis soil water consumption. Due to a lower precipitation storage efficiency, the reduction in soil water consumption in the normal season under DP was 27.10% and 15.57% under NT. In the two dry seasons (2015/2016 and 2016/2017), soil water consumption under DP was 16.29% and 13.00% higher than NT, respectively.

The N rate increased the soil water consumption, especially pre-anthesis, as well as total soil water consumption (Figs. 2B, 2D, 2F). The pre-anthesis soil water consumption and the total water consumption in the normal season was highest at 180 kg N ha^−1^ with NT and at 210 kg N ha^−1^ with DP, but the difference between 180 and 210 kg N ha^−1^ in both NT and DP was not significant. The pre-anthesis soil water consumption and the total water consumption under both NT and DP was highest at 150 kg N ha^−1^ in the 2015/2016 dry season and at 180 kg N ha^−1^ with NT and DP in the 2016/2017 dry season, but the difference in total soil water consumption in the 2016/2017 dry season between 150 and 180 kg N ha^−1^ in both NT and DP was not significant. These results indicate that the effective N rate is 180 kg N ha^−1^ in the normal season and 150 kg N ha^−1^ in the dry seasons, leading to the increase in soil water consumption.

### Effects of tillage and N rate on plant and soil N accumulation and consumption

#### Apparent soil N mineralization and N uptake efficiency

The average plant N accumulation in the normal season was 26.20–26.29%, and 18.10–19.50% higher than that in the two dry seasons (2015/2016 and 2016/2017). The NU_p_E in the normal season was 22.21–23.44% and 16.78–19.47% higher than that in the two dry seasons. The apparent N loss was reduced (Table 3) and this loss was affected by year, N rate, and the interactions of year × N rate (Table S1).

**Table 3:** Effect of tillage and N rate on soil N consumption and N uptake efficiency (NU_p_E) of 0–100 cm soil layer in 2014/2015, 2015/2016 and 2016/2017.

Year	N rate (kg ha^−1^)	Plant N accumulation (kg ha^−1^)		N_organic_ (kg ha^−1^)		N_loss_ (kg ha^−1^)		NU_p_E (%)	
		NT	DP	NT	DP	NT	DP	NT	DP
2014/2015	0	127.28 d	143.41 d	63.64 a	71.71 a	0	0	0	0
(Normal)	120	149.75 c	161.26 c	63.64 a	71.71 a	62.50 b	59.55 b	1.06 a	1.20 a
	150	160.98 b	169.54 b	63.64 a	71.71 a	73.57 b	71.94 b	1.00 a	1.08 ab
	180	165.25 a	180.27 a	63.64 a	71.71 a	71.28 b	70.18 b	0.89 ab	0.94 b
	210	161.86 ab	169.78 b	63.64 a	71.71 a	94.43 a	86.67 a	0.79 b	0.86 b
	Mean	153.02 B	164.85 A	63.64 B	71.71 A	75.45 A	72.09 A	0.94 B	1.02 A
2015/2016	0	112.01 c	116.78 d	56.01 a	58.39 a	0	0	0	0
(Dry)	120	126.16 a	134.52 b	56.01 a	58.39 a	80.96 c	72.99 c	0.93 a	0.97 a
	150	128.22 a	139.74 a	56.01 a	58.39 a	80.47 c	75.76 c	0.84 ab	0.90 ab
	180	120.48 b	136.75 ab	56.01 a	58.39 a	104.24 b	98.86 b	0.71 b	0.78 b
	210	118.98 b	125.35 c	56.01 a	58.39 a	123.65 a	118.65 a	0.57 c	0.65 c
	Mean	121.17 B	130.63 A	56.01 A	58.39 A	97.33 A	91.56 B	0.77 A	0.82 A
2016/2017	0	113.69 d	122.06 c	56.85 a	61.03 a	0	0	0	0
(Dry)	120	124.33 c	135.01 b	56.85 a	61.03 a	77.89 b	70.40 b	0.95 a	1.02 a
	150	135.22 ab	142.25 a	56.85 a	61.03 a	77.02 b	70.94 b	0.83 ab	0.90 ab
	180	141.76 a	146.89 a	56.85 a	61.03 a	83.59 b	82.02 b	0.75 b	0.79 b
	210	132.83 b	143.55 a	56.85 a	61.03 a	105.30 a	100.75 a	0.68 bc	0.70 bc
	Mean	129.57 B	137.95 A	56.85 A	61.03 A	85.95 A	81.03 B	0.80 A	0.85 A

**Note:**

No tillage (NT), and deep ploughing (DP). Apparent N mineralization (N_organic_), apparent N losses (N_loss_), and N uptake efficiency (NU_p_E), Means within the same column with different lowercase and capital letters were statistically significant at the critical probability levels of 0.05 by the LSD test. Lowercase and uppercase letters indicate comparisons among five treatments of N rate for each tillage methods and between two tillage methods, respectively.

The average plant N rate for DP was 7.73% higher than NT in the normal season, and 7.81% and 6.47% higher than NT in the two dry seasons. The NU_p_E for DP was 8.83% higher than NT in the normal season, and 7.74% and 6.38% higher than NT in the two dry seasons. However, the apparent N loss for DP was 4.46% lower than NT in the normal season, and 5.92% and 5.73% lower than NT in the two dry seasons. These results indicate that DP leads to higher NU_p_E, which promotes N accumulation in plants and reduces apparent N loss.

The N rate increased the plant N and apparent N loss, but reduced NU_p_E. The plant N rate for both NT and DP in the normal season was highest at 180 kg N ha^−1^, and the difference between 180 and 150 kg N ha^−1^ for NT was significant. The plant N rate for both NT and DP were highest at 150 kg N ha^−1^ in the dry season (2015/2016) and at 180 kg N ha^−1^ in the dry season (2016/2017), but the difference between 150 and 180 kg N ha^−1^ for both NT and DP in the dry season (2016/2017) was not significant. The apparent N loss for both NT and DP were highest at 210 kg N ha^−1^ in the normal and dry seasons, but the differences were not significant between 150 and 180 kg N ha^−1^ in the normal season and between 120 and 150 kg N ha^−1^ in the dry seasons. The NU_p_E for both NT and DP were highest at 120 kg N ha^−1^ in the normal and dry seasons, but the differences were not significant between 120 and 180 kg N ha^−1^ with NT in the normal season and between 120 and 150 kg N ha^−1^ for NT and DP in the dry seasons. These results indicate that the effective N rate is 180 kg N ha^−1^ in the normal season and 150 kg N ha^−1^ in the dry season, resulting in increased plant N accumulation with appropriate apparent N loss and NU_p_E.

#### Plant N accumulation and translocation

**Figure 3:**
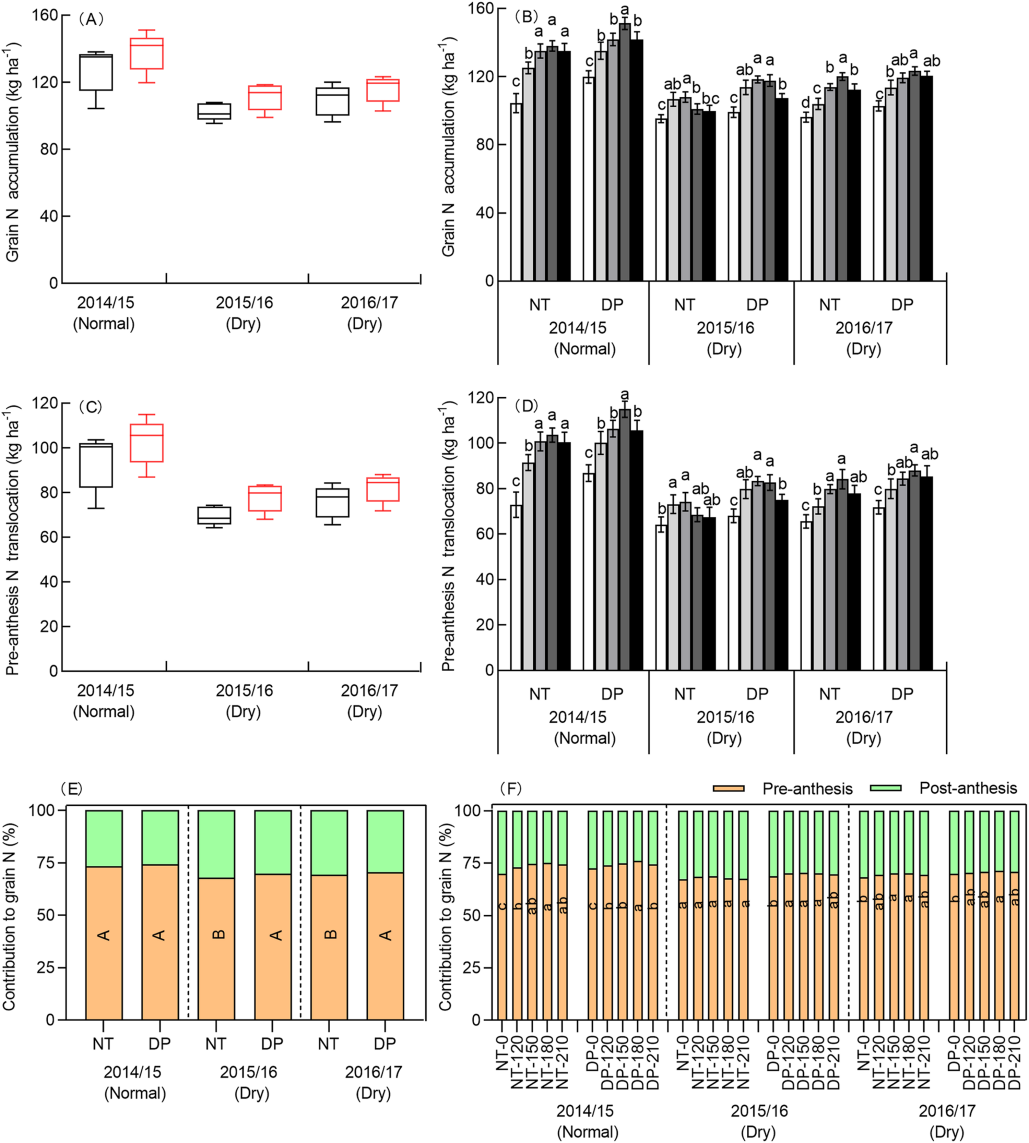
Effects of tillage and N rate on plant N accumulation and translocation in three seasons. No tillage (NT), and deep ploughing (DP). Grain N accumulation (A), pre-anthesis N translocation to grain (C) and contribution of pre-anthesis N translocation to grain (E) of two tillage in three seasons. Solid and red dashed lines represent median values, box boundaries represent the 25th and 75th percentiles, and bars and dots in or outside the boxes represent the 10th and 90th percentiles of all data. Grain N accumulation (B), pre-anthesis N translocation to grain (D) and contribution of pre-anthesis N translocation to grain (F) of two tillage among five N rates in three seasons. The means and SE of three replicates are presented. Lowercase and uppercase letters indicate comparisons among five treatments of N for each tillage method and between two tillage methods, respectively.

Grain N accumulation and pre-anthesis translocation were affected by year, tillage, N rate, and the interactions of all of these variables except tillage × N rate (Table S1). The effects of grain N accumulation and pre-anthesis N translocation were larger in the normal season than in the two dry seasons under NT and DT (Figs. 3A, 3C, 3E). Pre-anthesis N translocation in the normal season substantially increased by 32.12–35.00% and 23.57–25.46% more than in the two dry seasons. These results show that the normal season saw higher N accumulation and translocation in the winter wheat growing period, especially for pre-anthesis N translocation.

The effect of grain N accumulation and pre-anthesis N translocation was higher for DP than NT. Pre-anthesis N translocation for DP increased by 9.51% compared with NT in the normal season, and by 11.89% and 7.86% compared with NT in the two dry seasons. This result shows that DP largely promotes pre-anthesis N translocation to grain.

The N rate increased grain N accumulation and pre-anthesis N translocation to grain (Figs. 3B, 3D, 3F). Grain N accumulation and pre-anthesis N translocation to grain for both NT and DP in the normal season were highest at 180 kg N ha^−1^, but were highest at 150 kg N ha^−1^ in the 2015/2016 dry season and at 180 kg N ha^−1^ in the 2016/2017 dry season, but the difference of pre-anthesis N translocation between 150 and 180 kg N ha^−1^ was not significant in 2016/2017. These results indicate that the effective N rate is 180 kg N ha^−1^ in the normal season and 150 kg N ha^−1^ in the dry season, resulting in increased pre-anthesis N translocation and grain N accumulation.

#### Effect of tillage and N rate on yield, WUE, and NU_t_E

**Figure 4:**
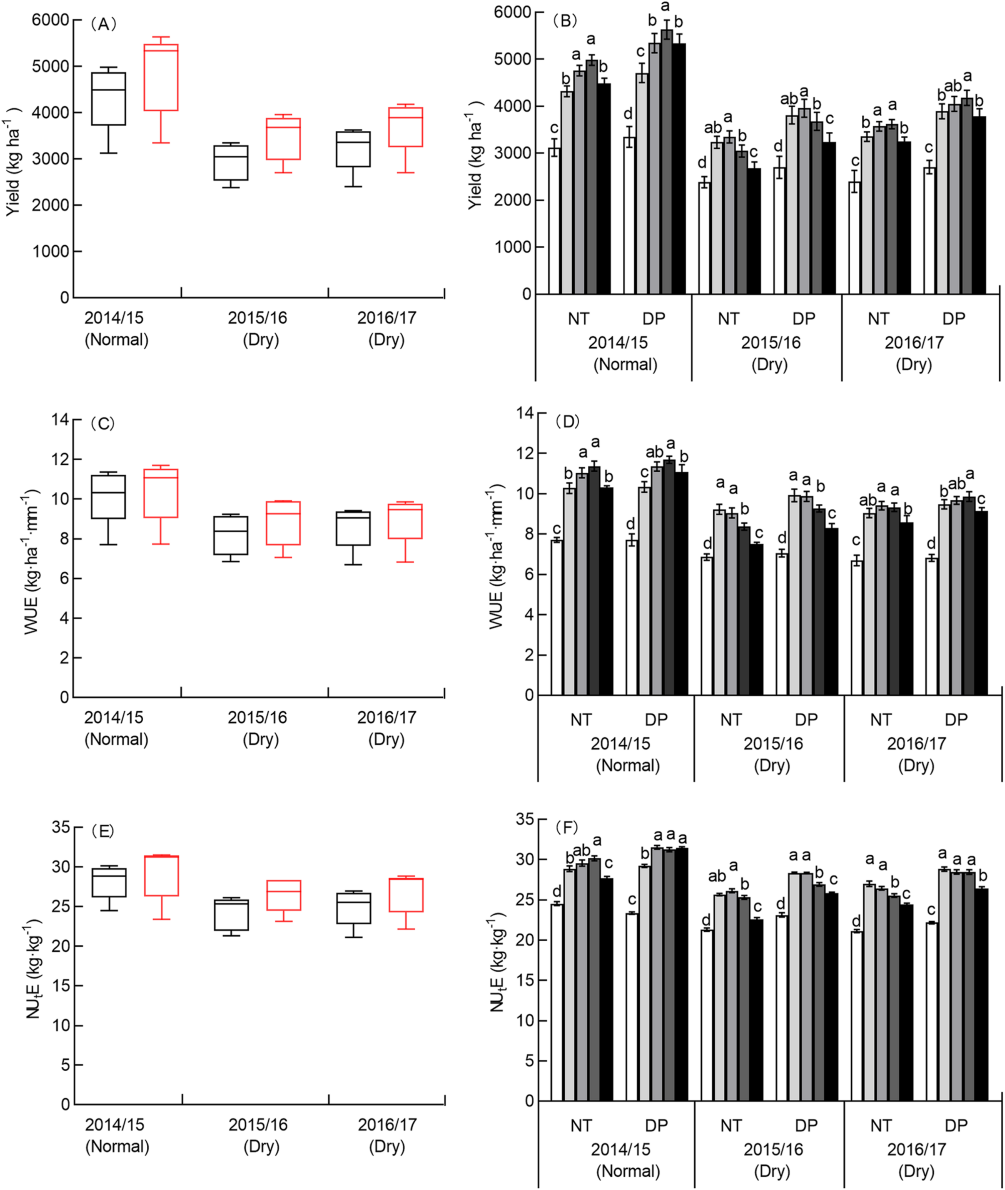
Effects of tillage and N rate on yield, WUE, and NU_t_E in three seasons. No tillage (NT), and deep ploughing (DP). Yield (A), WUE (C) and NU_t_E (E) of two tillage in three seasons. Yield (B), WUE (D) and NU_t_E (F) of two tillage at five different N rates in three seasons. The means and SE of three replicates are presented. Different lowercase letters indicate significant differences between treatments at *P* < 0.05 by the LSD test.

The effect of yield, WUE, and NU_t_E were affected by year, tillage, N rate, and the interactions of all of these variables except year × tillage and year × tillage × N rate on yield (Table S1). The average yield in the normal season was 40.10–47.37% and 30.92–33.78% higher than in the two dry seasons (Fig. 4A). The average WUE in the normal season was 17.49–23.67%, and 16.08–17.78% higher than the two dry seasons (Fig. 4C). The average NU_**t**_E in the normal season was 10.69–16.43%, and 9.27–13.08% higher than the two dry seasons (Fig. 4E). Compared with the two dry seasons, there was a substantial increase in yield during the normal season because of the higher water consumption.

The average yield for DP was 12.46% higher than NT in the normal season, and 18.29% and 14.92% higher than NT in the two dry seasons, respectively. The average WUE for DP was 2.88% higher than NT in the normal season, and 8.28% and 4.38% higher than NT in the two dry seasons, respectively. The average NU_**t**_E for DP was 4.21% higher than NT in the normal season, and 9.62% and 7.85% higher than NT in the two dry seasons, respectively. There was a reduction in the increasing rate for the yield, WUE, and NU_**t**_E in the normal season under DP due to the lower PSE_f_, as compared with the two dry seasons.

The N rate increased the yield, WUE, and NU_**t**_E (Figs. 4B, 4D, 4F). The yield for both NT and DP in the normal season was highest at 180 kg N ha^−1^, and the difference between 150 and 180 kg N ha^−1^ for DP was significant. The yield for both NT and DP were highest at 150 kg N ha^−1^ in the dry season (2015/2016) and at 180 kg N ha^−1^ in the dry season (2016/2017), but the difference between 150 and 180 kg N ha^−1^ in the dry season (2016/2017) for both NT and DP was not significant. The WUE for both NT and DP in the normal season was highest at 180 kg N ha^−1^. The WUE for both NT and DP were highest at 120 kg N ha^−1^ in the dry season (2015/2016), but at 150 kg N ha^−1^ for NT and at 180 kg N ha^−1^ for DP in the dry season (2016/2017); the difference in the dry season (2016/2017) between 150 and 180 kg N ha^−1^ for both NT and DP was not significant. The NU_**t**_E in the normal season was highest at 180 kg N ha^−1^ for NT, and at 150 kg N ha^−1^ for DP. The NU_**t**_E was highest at 150 kg N ha^−1^ for NT and at 120 kg N ha^−1^ for DP in the 2015/2016 dry season, and at 120 kg N ha^−1^ for both NT and DP in the 2016/2017 dry season, but the difference in the two dry seasons between 120 and 150 kg N ha^−1^ for both NT and DP was not significant. These results indicate that the effective N rate is 180 kg N ha^−1^ in the normal season and 150 kg N ha^−1^ in the dry season, leading to higher yield and N efficiency.

#### Correlations among soil precipitation storage during the summer fallow season, soil water consumption, and yield

**Figure 5:**
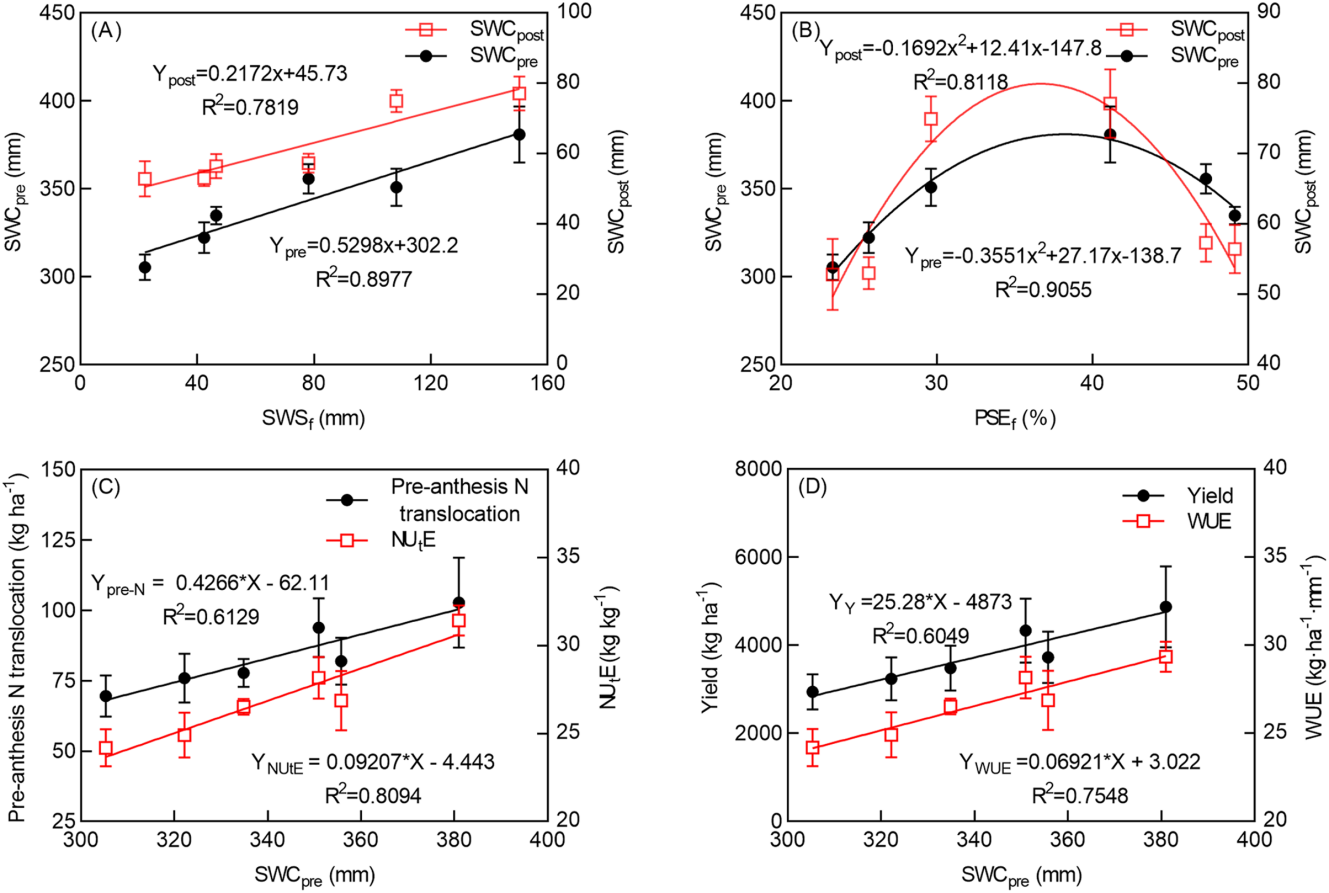
Relationships between soil precipitation storage amount and efficiency during summer follow seasons and soil water consumption and yield (*n* = 6). The means and SE of three seasons are presented by the average of five different N rates under two tillage.

The SWS_f_ was significantly and positively related to SWS_pre_ and SWS_post_ (Fig. 5A). With a 1 mm increase in SWS_f_, the SWS_pre_ and SWS_post_ increased by 0.5298 and 0.2172 mm, respectively. There was a quadratic relationship between PSE_f_ and SWS_pre_ (R^2^ = 0.9055) and SWS_post_ (R^2^ = 0.8118) (Fig. 5B) across the experimental seasons, and the decreasing SWS with the increasing PSE_f_ may have been caused by extremely low precipitation during the summer fallow season, leading to higher PSE_f_ (2014/2015). However, SWS due to low precipitation is not enough to satisfy the needs of seed germination and tiller production, or to reduce SWS and yield production indirectly. In summary, SWS_f_ and PSE_f_ have a stronger relationship with pre-anthesis soil water consumption, and SWC_pre_ significantly affected pre-anthesis N translocation and NU_t_E (Fig. 5C). An increase of 1 mm in SWC_pre_ increased grain yield by 25.28 kg ha^−1^, WUE by 0.069 kg ha^−1^ mm^−1^, and NU_t_E by 0.029 kg kg^−1^ (Fig. 5D).

#### Response of yield, WUE, and NU_t_E to N rate under NT and DP

**Figure 6:**
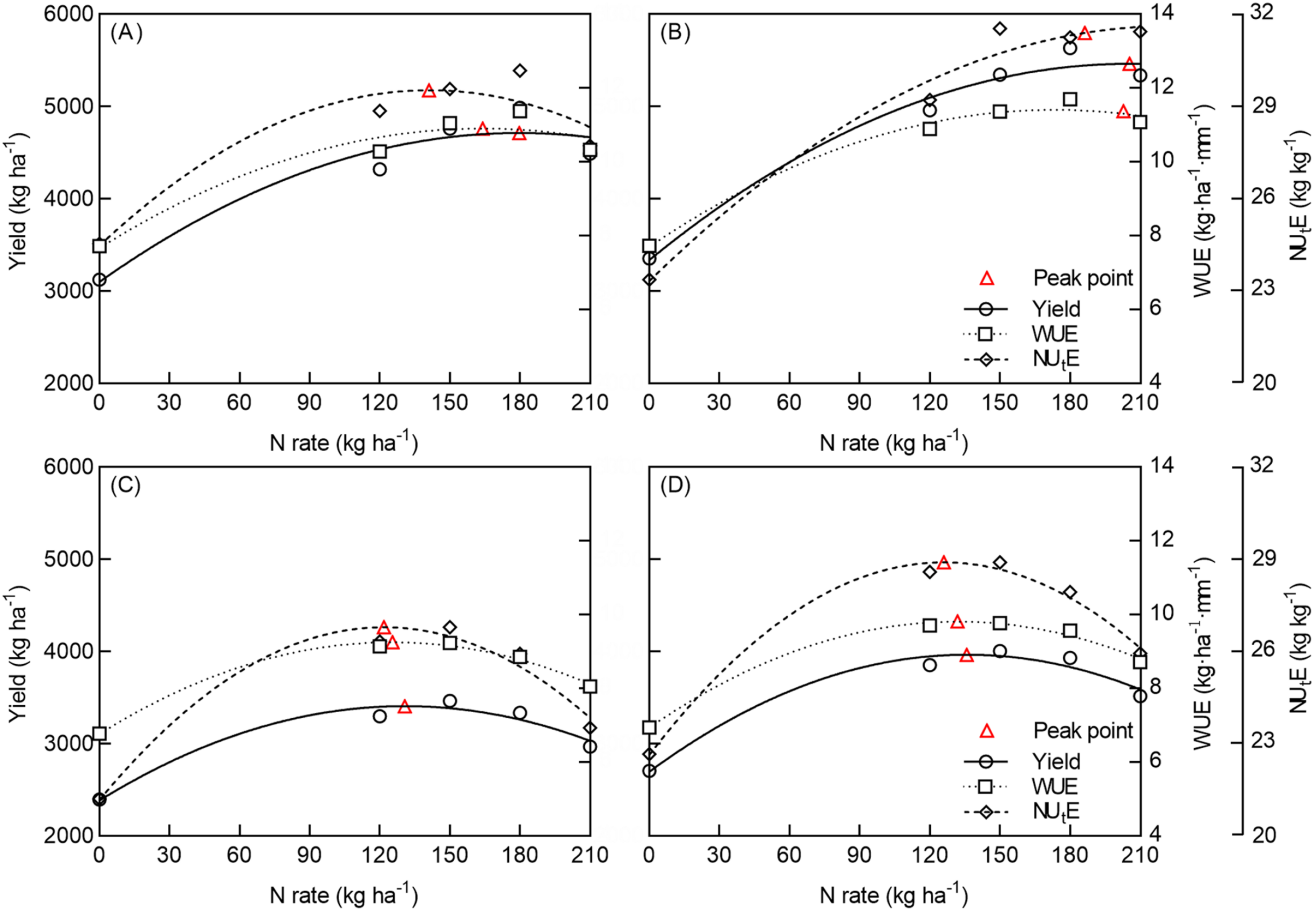
Response of yield, WUE, and NU_t_E to N rate under two tillage methods in normal and dry seasons (*n* = 5). No tillage (NT) and deep ploughing (DP). Responses of no tillage in normal (A) and dry (C) seasons, and responses of deep tillage in normal (B) and dry (D) seasons. The red triangles represent the peak points of each fitting curves. The red dotted line represents the N rate under deep ploughing in the same peak points of yield, WUE, and NU_t_E under no tillage in normal and dry seasons.

Below a specific N application rate, the yield, WUE, and NU_t_E under NT and DP increased with an increasing N rate; above a specific N application rate (red symbol, the optimal N rate), it then decreased gradually in both the normal and dry seasons (shown in Fig. 6).

In the normal season, the yield, WUE, and NU_t_E for NT reached their highest points when the N application rate was 179, 164, and 142 kg ha^−1^, respectively; and the yield, WUE, and NU_t_E for DP reached the highest points when the N application rate was 205, 202, and 186 kg ha^−1^, respectively. In the dry season, the yield, WUE, and NU_t_E for NT reached the highest points when the N application rate was 131, 125, and 122 kg ha^−1^, respectively; the yield, WUE, and NU_t_E for NT reached the highest points when the N application rate was 136, 132, and 126 kg ha^−1^, respectively.

Replacing NT with DP for the same highest point of yield, WUE, and NU_t_E, could reduce the N rate by 11.49–53.34% in the normal season and 40.97–65.07% in the dry seasons.

## Discussion

### Tillage and precipitation in the summer fallow season strongly affect SWS_f_ and SWC_pre_

The distribution of summer fallow precipitation varies greatly among different regions. In the Mediterranean climate region, precipitation during the summer fallow season accounts for less than 30% of the annual total, whereas it accounts for approximately 60–70% of the annual total on the Loess Plateau. During 2015–2016, 24.48% of the annual precipitation occurred in the fallow season, but 70.76% of the annual precipitation occurred during the summer fallow season in 2014–2015 (Fig. 1). Therefore, to ensure high and stable yields in the drylands of the Loess Plateau, it is necessary to store water during the summer fallow season to meet the water requirements of the crops during later growing stages (Zhang et al., 2008; Wang & Shangguan, 2015).

Sufficient pre-sowing soil moisture is a prerequisite for high wheat yield in drylands, whereas low pre-sowing soil moisture reduces yield (Wang et al., 2016). Studies have shown that 47% of wheat yield is dependent on soil storage before sowing (Huang et al., 2003). Tillage in the summer fallow season can significantly improve the precipitation utilization rate, SWS, and WUE at sowing (Xue et al., 2018), which is consistent with the results of our study. Our study shows that tillage (DP) in the summer fallow season significantly increases SWS at sowing and PSE_f_ in the summer fallow season; PSE_f_ in 2014–2015, 2015–2016, and 2016–2017 was 41.13%, 49.13%, and 47.28%, respectively, and was higher in the dry season than in the normal season (Table 2). PSE_f_ has a significant and positive relationship with grain yield (Sun et al., 2018), but this study shows that higher PSE may lead to lower water consumption and thus lower yield, while deep ploughing increases PSE, which is also a possible negative effect of deep ploughing (Fig. 5). Soil water consumption is enhanced by increasing the N rate, and the effect is more significant for soil water consumption from sowing to anthesis as compared with anthesis to maturity (Khan et al., 2020). Our results show that SWS_f_ is significantly and positively related to SWC_pre_, with a 1 mm increase in SWC_pre_ increasing grain yield by 25.28 kg ha^−1^. PSE_f_ and SWC_pre_ showed a quadratic linear correlation reflecting the significantly lower precipitation during the summer fallow period.

Tillage can improve SWS at sowing because of decreasing soil bulk density and improved soil porosity (Al-adawi & Reeder, 1996; Schiettecatte et al., 2005). However, conflicting results have been reported, with some showing that short-term tillage during the fallow season had a slight effect on soil properties, NT increased soil moisture more effectively than SS, and SWS at sowing improved by 3.0% under NT compared with SS (Wang et al., 2009; Xue et al., 2017). However, we found that under NT, SWS before sowing, precipitation storage efficiency of summer fallow, and WUE were all lower than under DP (Table 2, Fig. 4), suggesting that these parameters caused a low yield. This could also be due to soil compaction, which is unfavorable for both infiltration and storage of precipitation in the summer fallow, and for the management and elimination of weeds. This is consistent with the results of Camara, Payne & Rasmussen (2003). DP is an effective tillage method for improving SWS and water consumption; thus, DP improved WUE and yield, and increased yield by 12.46–18.29% and WUE by 2.88–8.28% (Fig. 4), which is consistent with the results obtained in the current study (Sun et al., 2018).

### Tillage and N rate strongly effect pre-anthesis N translocation

Tillage practices can modify the soil environment, improve porosity, increase disintegration of aggregates, and mix plant materials deeper into the soil, thereby increasing crop biomass and soil contact (Aziz, Mahmood & Islam, 2013). Reducing nitrate N leaching through optimal tillage can affect the absorption of N by plants, which is important for soil, water, and other environmental resource protection (Wang et al., 2015). Tillage can affect soil mineralization by changing soil physical properties (Wang et al., 2010; Mu et al., 2016). In NT systems, N immobilization and loss are higher, which contributes to decreases in soil mineral N (Ruisi et al., 2016). In ploughed systems, the mineralization rate of organic N is higher than in NT systems. Moreover, NT increases N immobilization and contributes to reducing soil N availability as compared with those under tillage because crop residues are deposited on the soil surface under no-tillage conditions (Giller et al., 2009). Our study shows that DP in the summer fallow season increased the N mineralization and N absorption efficiency in the normal seasons, reduced the apparent N loss in the dry seasons, and improved the utilization of N in different seasons according to the source and location of N, compared with NT (Table 2). Tillage practices cause leaching of NO_3_^−^-N in the soil by offering a shorter diffusion path and a larger surface contact area (Matthews et al., 2000).

Increased yields may also be because of more efficient use of soil water and available nitrogen due to tillage practices. Tillage during the summer fallow season increases pre-anthesis N translocation and yield (Sarker et al., 2017; Liang et al., 2019). The improvement in plant N under tillage was largely caused by an increase in the N available in the soil (Soon et al., 2007; Soon, Brandt & Malhi, 2006; Ruisi et al., 2016). This study showed a similar result: DP during the summer fallow season could promote plant N accumulation at maturity, promote N translocation, and lead to higher N uptake and utilization efficiency (Fig. 3). Combined with soil N mineralization and apparent N loss, applying DP during the summer fallow season increased N mineralization in the soil, reduced or maintained the apparent N loss and increased uptake efficiency. Thus, more N was transferred from the soil to the aboveground plant, and the N utilization efficiency aboveground was higher resulting in higher N accumulation in the plant.

### Adjusting N rate according to yield and efficiency in different precipitation seasons

The availability of water at sowing is the most crucial factor for N uptake and utilization on the Loess Plateau (Fu et al., 2014). Jan, Amanullah & Hussain (2016) found that adjusting the N rate according to precipitation could produce a higher wheat yield compared with baseline application rates. Some studies identified optimal N rates by finding the peak points of the curves for the N rate and each related indicator (such as environmental cost, N uptake, water and N utilization, and yield). Yield is often used to optimize the N rate (Chen et al., 2014; Wang et al., 2017) because it is the most relevant concern for both farmers and government agencies as it impacts farmer livelihood and national food security.

An optimal N rate is necessary to achieve high yield, however there is a limit to how much N crops can absorb, so the N rate should be adjusted according the maximum N accumulation of crops (Zhong, Ju & Zhang, 2006). N use efficiency can also be used as a reference trait to estimate suitable N rates (Liang et al., 2008; Zhang et al., 2012; Wang et al., 2014). Yu et al. (2021) optimized the N rate of different precipitation seasons. In their study, the results showed that maximum yields were obtained when the N rates were 150 and 180 kg N ha^−1^ in the dry and normal seasons, respectively. This is consistent with the results of our study, which used field data (Fig. 4) and the optimal N rates were determined based on maximum yield and efficiency. In the normal season, the optimal N rate was 142–179 kg ha^−1^ for NT and 186–205 kg ha^−1^ for DP; in the dry season, the optimal N rate was 122–131 kg ha^−1^ for NT and 126–136 kg ha^−1^ for DP (Fig. 6). For the same highest point of yield, WUE, and NU_t_E, replacing NT with DP could reduce the N rate by 11.49–53.34% in the normal seasons and 40.97–65.07% in the dry seasons (Fig. 6). This may be because winter wheat under DP can efficiently use nitrogen in the dry seasons because of the higher apparent N mineralization and NU_p_E (Ruisi et al., 2016), which reduces the N rate significantly. Therefore, applying DP during the summer fallow season in place of NT can improve yield, water, and N use efficiencies and even reduce N application rates for the same yield level. As such, it is feasible to optimize N rates according to yield, water, and N use efficiencies under different precipitation conditions.

## Conclusions

We determined that deep ploughing during the summer fallow season improves soil water storage and precipitation storage efficiency, which have significant linear and quadratic correlations with pre-anthesis soil water consumption. DP also promotes plant nitrogen translocation, and increases yield of winter wheat compared with NT. A 1 mm increase in pre-anthesis soil water consumption could increase grain yield by 25.28 kg ha^−1^. DP could reduce the N rate by 11.49–53.34% in the normal seasons and 40.97–65.07% in the dry seasons compared to the same highest point of yield, WUE, and NU_t_E under NT. Deep ploughing combined with an optimal N rate based on precipitation year type can improve the utilization efficiency of water and fertilizers, maintain productivity, and promote the sustainable development of resources and the environment.

## Supplemental Information

10.7717/peerj.14153/supp-1 Supplemental Information 1 The result of all the data involved in the figures and tables. The specific values of soil water consumption, plant nitrogen accumulation and translocation, grain yield and water and nitrogen use efficiency of dryland winter wheat under different precipitation years, tillage methods and nitrogen application rates.

10.7717/peerj.14153/supp-2 Supplemental Information 2 Tillage practice at summer fallow of no tillage (NT, A) and deep ploughing (DP, B).

10.7717/peerj.14153/supp-3 Supplemental Information 3 Precipitation and temperature in 2014/2015, 2015/2016 and 2016/2017.

10.7717/peerj.14153/supp-4 Supplemental Information 4 The ANOVA of soil water consumption, N balance and utilization. Y, year; T, tillage; N, N rate. NUpE, N-uptake efficiency, NUtE: Nitrogen-utilisation efficiency.

## Additional Information and Declarations

### Data Availability

The following information was supplied regarding data availability: The raw measurements are available in the Supplemental File.
