# Supplementary figures and images for: Deep ploughing in the summer fallow season and optimizing nitrogen rate can increase yield, water, and nitrogen efficiencies of rain-fed winter wheat in the Loess Plateau region of China
Source: PeerJ. 2022 Oct 7;10:e14153. doi: 10.7717/peerj.14153 (PMC9549900; doi:10.7717/peerj.14153)

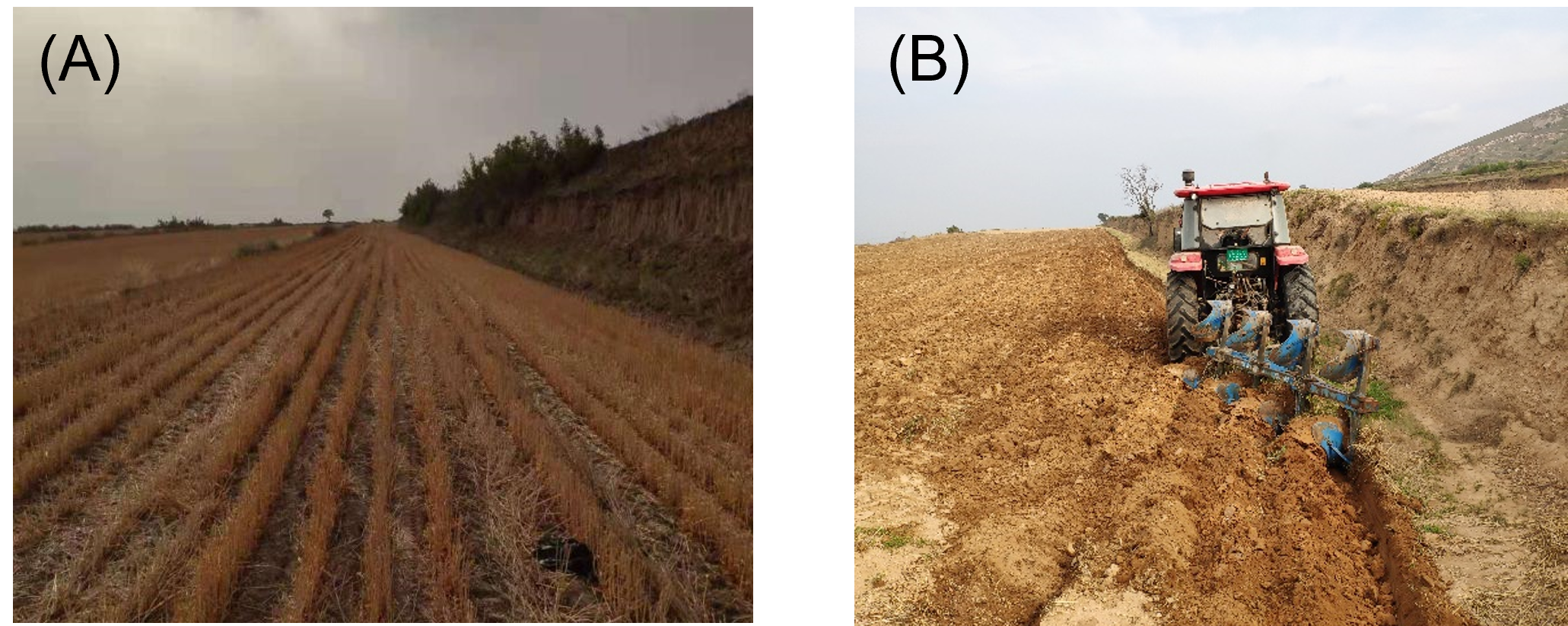

Supplement: Supplemental Information 2 [file peerj-10-14153-s002.png]

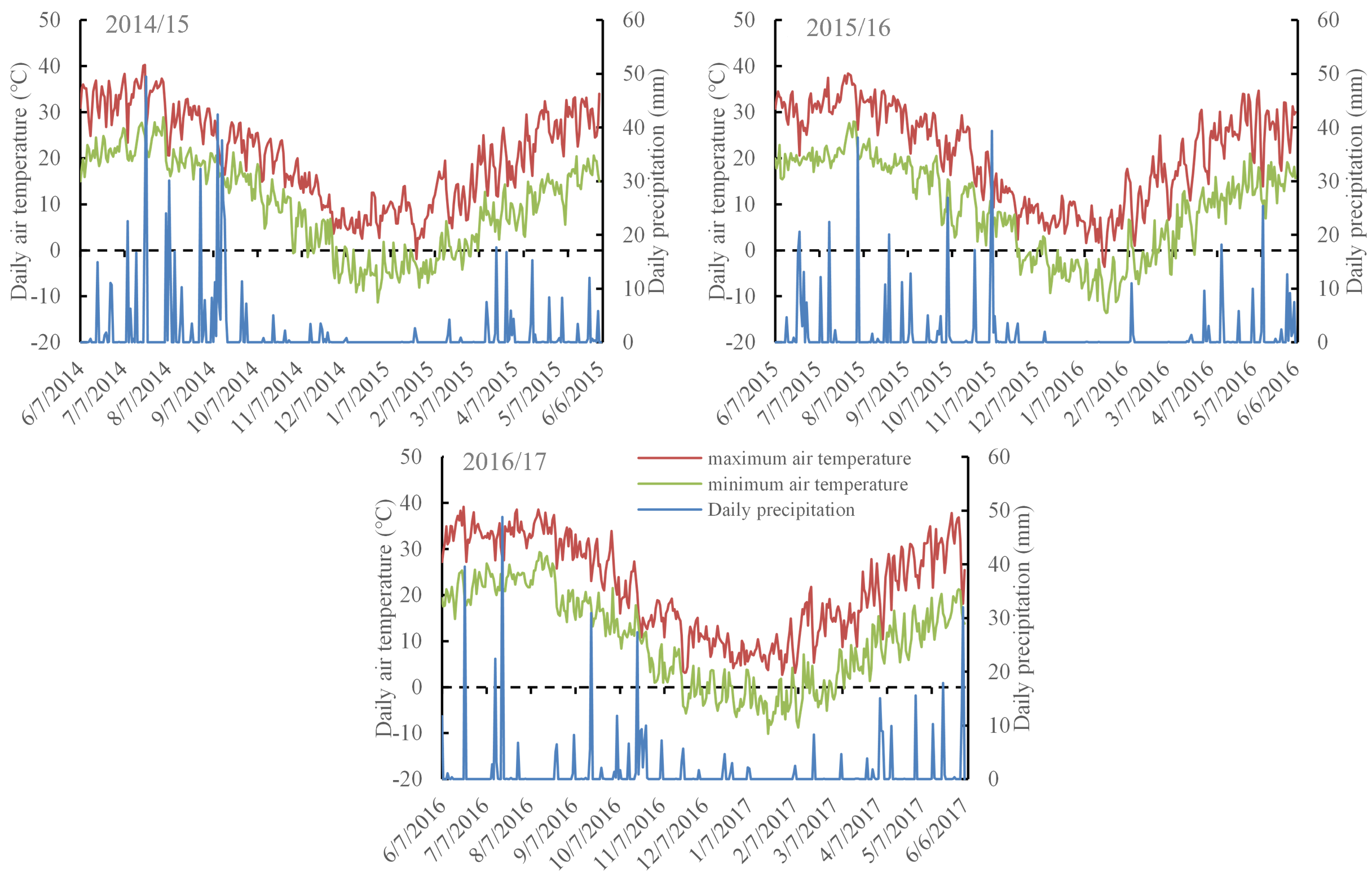

Supplement: Supplemental Information 3 [file peerj-10-14153-s003.png]
