# Supplementary material for: Deep ploughing in the summer fallow season and optimizing nitrogen rate can increase yield, water, and nitrogen efficiencies of rain-fed winter wheat in the Loess Plateau region of China
Source: PeerJ. 2022 Oct 7;10:e14153. doi: 10.7717/peerj.14153 (PMC9549900; doi:10.7717/peerj.14153)
Supplement: Supplemental Information 4 — Y, year; T, tillage; N, N rate. NUpE, N-uptake efficiency, NUtE: Nitrogen-utilisation efficiency. [file peerj-10-14153-s004.docx]

| **Table S1 The ANOVA of soil water consumption, N balance and utilization.** | | | | | | | | |
| --- | --- | --- | --- | --- | --- | --- | --- | --- |
| Traits |  | Y | T | N | Y×T | Y×N | T×N | Y×T×N |
|  | df | 2 | 1 | 4 | 2 | 8 | 4 | 8 |
| Soil water consumption | Pre-anthesis | 0.000 | 0.000 | 0.000 | 0.001 | 0.000 | 0.045 | 0.030 |
|  | Post-anthesis | 0.000 | 0.000 | 0.000 | 0.179 | 0.000 | 0.048 | 0.334 |
|  | Total | 0.000 | 0.000 | 0.000 | 0.000 | 0.000 | 0.030 | 0.000 |
| Soli nitrogen | Apparent N losses | 0.002 | 0.297 | 0.000 | 0.975 | 0.045 | 0.982 | 1.000 |
| Plant N accumulation and remobilisation | Grain N | 0.000 | 0.000 | 0.000 | 0.000 | 0.000 | 0.000 | 0.000 |
|  | pre-anthesis N translocation | 0.000 | 0.000 | 0.000 | 0.000 | 0.000 | 0.000 | 0.000 |
|  | contribution to grain N | 0.008 | 0.000 | 0.002 | 0.000 | 0.000 | 0.194 | 0.000 |
| Yield | Yield | 0.000 | 0.000 | 0.000 | 0.812 | 0.000 | 0.041 | 0.888 |
| WUE | WUE | 0.000 | 0.000 | 0.000 | 0.000 | 0.000 | 0.000 | 0.000 |
| N uptake and utilization | NU_p_E | 0.000 | 0.000 | 0.000 | 0.000 | 0.000 | 0.000 | 0.000 |
|  | NU_t_E | 0.000 | 0.000 | 0.000 | 0.000 | 0.000 | 0.000 | 0.000 |
| Y, year; T, tillage; N, N rate. NU_p_E, N-uptake efficiency, NU_t_E: Nitrogen-utilization efficiency. | | | | | | | | |
